# Contribution to regulatory science and a next challenge of the Japanese Environmental Mutagen Society (JEMS)

**DOI:** 10.1186/s41021-016-0040-1

**Published:** 2016-09-01

**Authors:** Yoshifumi Uno

**Affiliations:** Safety Research Laboratories, Sohyaku. Innovative Research Division, Mitsubishi Tanabe Pharma Corporation, 2-2-50, Kawagishi, Toda, Saitama, 335-8505 Japan

**Keywords:** Genotoxicity, Risk assessment, Guideline, Weight of evidence, Mechanism of action

## Abstract

Many members of The Japanese Environmental Mutagen Society (JEMS) have significantly contributed to guidelines on chemical genotoxicity. The guidelines have been useful for the hazard identification and risk assessment of genotoxic chemicals. However, risk assessors and developers of drugs and other commercial products might eliminate beneficial chemicals from further development simply based on positive results of genotoxicity testing. Experts in the field of genotoxicity should better characterize the biological significance of genotoxicants and more correctly assess human risk. I hope that one of the next challenges undertaken by JEMS will be to assess the human risk of genotoxic chemicals more correctly based on the precise analysis of their mechanisms of action.

The Japanese Environmental Mutagen Society (JEMS) was established in 1972; its main purpose is to investigate environmental mutagens that may affect public health. Therefore, one of the concerns of JEMS is to characterize the genotoxicity, including mutagenicity, of chemicals to which humans are exposed. A large number of relatively simple in vitro and in vivo test methods have been developed to detect genotoxic compounds and several of these are internationally standardized as test guidelines by the Organisation for Economic Co-operation and Development (OECD) [1–13]. In addition, strategic guidelines (or international consensus papers) have been published to describe how chemical genotoxicity should be evaluated and regulated to reduce the risk for humans. One example is the S2 guidelines of the International Conference on Harmonization of Technical Requirement for Registration of Pharmaceuticals for Human Use (i.e., ICH-S2 guidelines) [14–16]. Many members of JEMS have significantly contributed to such guidelines, and currently most new chemicals are evaluated using such test methods because of more strict regulation of genotoxicants than before.

These guidelines have been very useful for the hazard identification and risk assessment of genotoxic chemicals. However, risk assessors and developers of drugs and other commercial products might eliminate chemicals from further development simply based on positive results of genotoxicity testing. For example, the bacterial reverse mutation test (i.e., the Ames test) is generally used in the early screening of pharmaceutical candidates, and many drug developers believe that positive results in this assay necessitate the withdrawal of the candidate from further development. Although experts in the field of genotoxicity know that Ames-positive results do not always mean a risk to humans, discarding positive candidates is thought to be cost-effectiveness, i.e., extensive efforts would be required to demonstrate that the Ames-positive results were not relevant to human. This strategy is also preferable from the viewpoint of pharmaceutical regulation. However, might not this strategy also reduce the likelihood of developing useful pharmaceuticals? Experienced medicinal chemists avoid synthesizing pharmaceuticals that have known genotoxic substituents and/or possibly genotoxic structure, thus narrowing the chemical-space of new pharmaceutical candidates. Historically, some genotoxic pharmaceuticals have been accepted for medical use by regulatory agencies based on a risk-benefit consideration but the indications are largely limited to the treatment of cancers or infectious diseases. Since there are many other life-threating and/or intractable diseases, the elimination of genotoxicity from chemical-space might be a disadvantage for patients with serious diseases. I believe that this is a very important issue, which genotoxicity experts should carefully consider.

How can experts better characterize the biological significance of genotoxicants and more correctly assess human risk? A general approach for examining chemicals is to use a battery of genotoxicity tests that can detect different kinds of genotoxicity, i.e., use a weight of evidence (WoE) approach. For example, the ICH-S2(R1) guideline [16] requires two types of in vivo genotoxicity tests when a chemical shows a positive result in an in vitro genotoxicity test using mammalian cells. However, two negative in vivo test results might be insufficient in terms of concluding that a chemical poses absolutely no genotoxicity risk to humans. This is true because in vivo genotoxicity test methods are generally insensitive compared to in vitro ones. Negative results in rodent carcinogenicity bioassays with rats and mice would substantially support a conclusion that the genotoxic active response was not relevant, but the testing of all genotoxic agents for cancer induction in rodents is just not feasible. So, how might experts approach this issue? The first issue of “Genes and Environment”, published ten years ago, included my report, in which suggesting that one approach to understanding the role of genotoxicity in carcinogenesis would be the precise analysis of the genotoxic mechanisms of action (MoA) [17]. And here, I reiterate the same suggestion to resolve the issue mentioned above by introducing two approaches for analyzing the MoA and for assessing human risk based on the MoA.

One of the approaches is based on the availability of new technologies. My colleagues and I have integrated the DNA adductome approach, i.e., identification of the types and frequency of chemically-induced DNA adducts [18] to assess the DNA-damaging capability of in vitro micronucleus (MN) test-positive chemicals. In addition to the Ames test, the in vitro MN test is generally used for the screening of chemicals for genotoxicity, but it often produces false-positive results [19]. When a positive MN result is obtained for a chemical, the first consideration in terms of human risk is whether the chemical reacted with DNA directly or indirectly; the former would indicate human risk while the latter would potentially indicate the existence of a no-adverse-effect level. Thus, DNA adductome analysis is a useful method for determining MoA. In the experiments using 9 chemicals positive in the in vitro MN test with Chinese hamster lung cells, 6 carcinogens formed DNA-adducts while 3 non-carcinogens did not [20]. These findings indicate that DNA adductome analysis can provide useful information about the potential of a positive in vitro result to pose a human risk.

Another approach is case-by-case that considers the chemical and/or biological (pharmacological in pharmaceuticals) properties of a chemical in a WoE approach. As a case study, MP-124, a novel poly(ADP-ribose)polymerase-1 (PARP-1) inhibitor, is being developed as a neuroprotective agent against acute ischemic stroke [21, 22], and my colleagues and I hypothesized during the early phase of development that the pharmacological property might produce a genotoxic event because PARP-1 is a key enzyme involved in the repair of DNA damage [23–27]. Therefore, the genotoxicity of MP-124 was carefully investigated by using the WoE approach [28]. The compound was positive in the in vivo immature erythrocyte MN test using male rats treated by intravenous infusion [28], and the MoA of the positive finding was investigated. Since MP-124 inhibits PARP-1 competitively and the endogenous competitor is nicotinamide adenine dinucleotide (NAD) [21, 22], Yamamura et al. examined whether or not co-treatment with nicotinic acid, the precursor of NAD, to rats could inhibit the induction of MN by MP-124; co-treatment clearly inhibited MN induction [28]. They also examined if co-treatment of cyclophosphamide and nimustine, both reference genotoxicity positive-controls, with NAD under the same experimental conditions altered their ability to induce MN, but no inhibition was observed [28]. These results indicate that the ability of MP-124 that induced MN in vivo is related to its pharmacological properties and therefore, this mechanism is expected to result in a threshold for MN induction. This understanding allows for the establishment of a safe margin of exposure for the therapeutic use of MP-124. Although this is a case study, a similar approach can be applied to other cases if genotoxicity experts well understand the chemical/biological properties of the chemical of interest in advance, and hopefully before starting the development of new pharmaceuticals.

As members of JEMS, we know that there are still many issues that should be investigated like the above examples, but it seems that the present genotoxicity test guidelines are considered by those who are not experts on genotoxicity to be sufficient for assessing the genotoxic risk of chemicals. To bridge any discordance in understanding between us and the rest of the scientific and regulatory community, I think that we should more actively inform the non-experts of our update that is more logical for their satisfaction beyond the simple testing results on chemical genotoxicity. Therefore, I hope that one of the next challenges undertaken by JEMS will be to assess the human risk of genotoxic chemicals more correctly based on the precise analysis of their MoA.

## Abbreviations

ICH: the International Conference on Harmonization of Technical Requirement for Registration of Pharmaceuticals for Human Use
JEMS: The Japanese Environmental Mutagen Society
MN: micronucleus
MoA: mechanisms of action
NAD: nicotinamide adenine dinucleotide
OECD: The Organisation for Economic Co-operation and Development
PARP: poly(ADP-ribose)polymerase
WoE: weight of evidence
